# Supplementary material for: Development and User Experiences of a Novel Virtual Reality Task for Poststroke Visuospatial Neglect: Exploratory Case Study
Source: JMIR XR Spat Comput. 2025 Aug 15;2:e72439. doi: 10.2196/72439 (PMC12671316; doi:10.2196/72439)
Supplement: Multimedia Appendix 1 [file xr-v2-e72439-s001.docx]

# Supplementary Information and Materials

**Figure 1 Section A: Unity 3D rendered scene of the virtual environment**


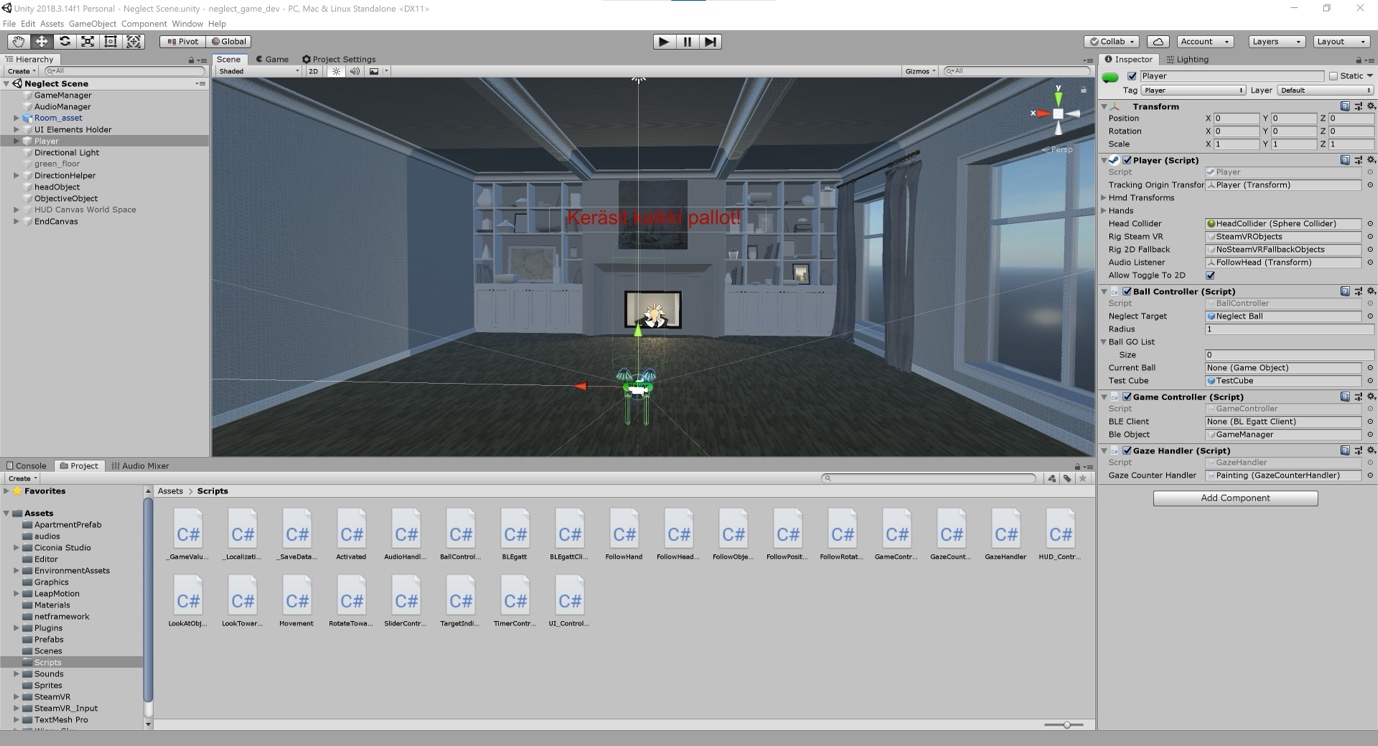


*Note.* The Unity 3D rendered scene of the virtual environment. The scene displays a room with a fireplace over which a painting is hung to signify the central gaze point for initiating ball appearance. Within this environment, a ball appears 15° to the left within a 30° horizontal and 50° vertical plane. The scene also includes windows, a ceiling, and additional objects to enhance user immersion. The image shows the Unity interface with the virtual room, scripts, and assets used to create and control the VR experience.

**Figure 2 Section B: Display of the in-task application menu**


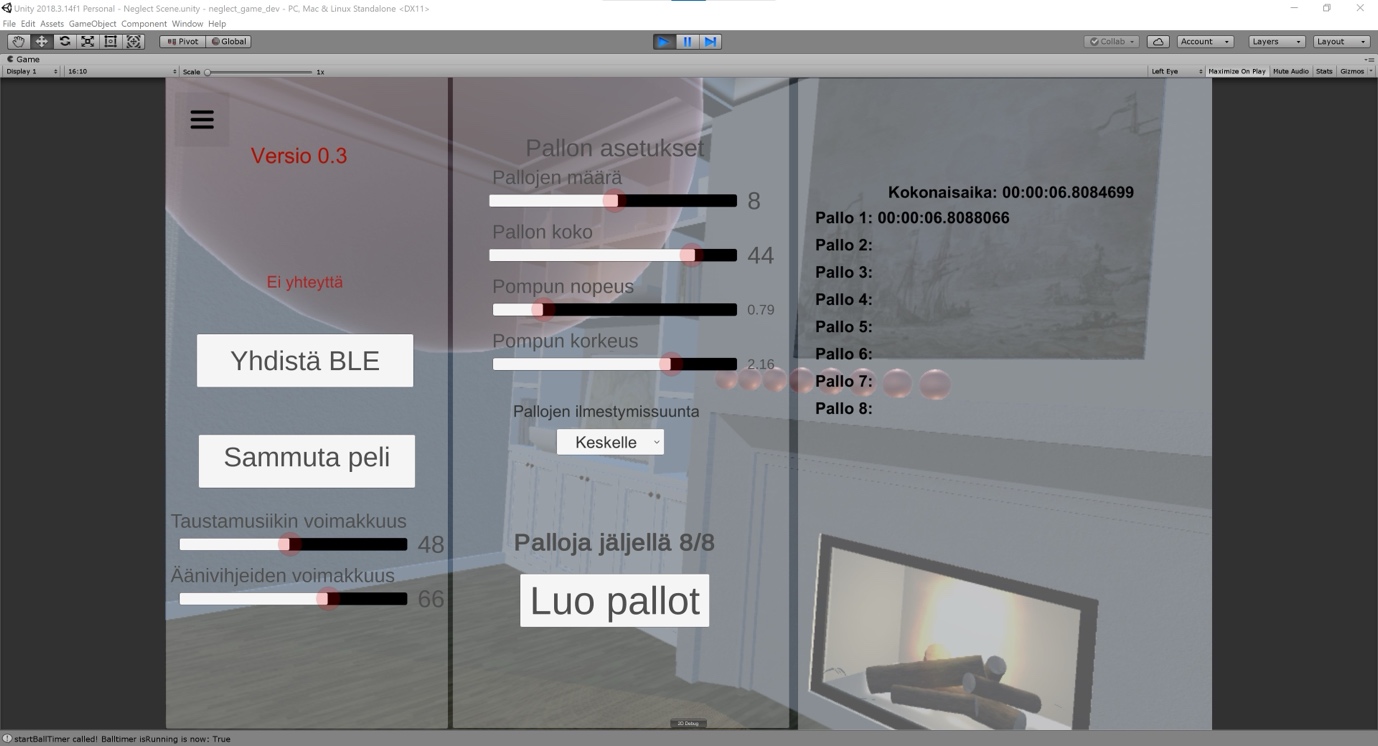


*Note***.** A display of the in-task application menu used by physiotherapists to configure settings before each trial. Note this menu is illustrative and not representative of the precise parameters used during sessions. The menu, in the Finnish language, includes details such as 'Version 0.3', and options such as 'Connect to Bluetooth', and 'Quit Application', as well as controls for the 'Volume of Background Music' and 'Volume of Audio Cues'. The 'Ball Settings' section allows customization of the number of trials, ball size, bounce speed, bounce height, and the balls' direction of appearance. The final option, 'Create Balls', initiates the display of balls for the trial. The physiotherapists interact with this menu using a mouse and keyboard with the PC-based system, adjusting settings to tailor the difficulty and assistance level of each trial for the patient.

**Table 1 Section A.** The questions from the questionnaire are shown in Table S1.

| Questionnaire, number | Question | Domain |
| --- | --- | --- |
| **TAM^a,b^**    1    2    3 | I think the system is easy to use    Learning to use the system is not a problem    I enjoyed using the system | Usability    Usability    Usability |
| 4 | I would like to use the system in the future if I had the opportunity | Usability |
| **EIGQ^c,b^**    5    6 | Learning to use this VR task was easy    Was learning the task difficult? | Engagement      Engagement |
|  |  |  |
| **SAQ^d,b^**    7 | I would feel safe using this system as a patient | Safety |
|  |  |  |

*Note.*

^a^ TAM: Technology Acceptance Model Questionnaire

^b^ Fully disagree to fully agree, 1–5, scored individually

^c^ EIGQ: Engagement in In-Game Questionnaire

^d^ SAQ: Safety Attitudes Questionnaire

**Table 2 Section B.** Summaries of the two patient profiles.

| **Patient A** | **Patient B** |
| --- | --- |
| Male | Female |
| 46 years old | 37 years old |
| Stroke description:    Left-sided hemiparesis^a^, visuospatial neglect.  Received intervention 1 year after the stroke (in 2022). | Stroke description:    Left-sided hemiplegia^b^, neglect symptoms.  Received intervention 4 years after the stroke (in 2022). |
| Brief description of patient functional capacity:  “Slight active movement on the left arm, able to stand and take a few steps with strong support. Generally, moves with an electric wheelchair (and steers this with the right-hand) or manual wheelchair. Uses his right hand in everyday life and needs help with dressing and cooking.” | Brief description of patient functional capacity:  “Walking with a walking stick, occasional spasticity in the left upper limb which functions slightly as a support hand.” |

^a =^ Left-sided hemiparesis is characterized by weakness or the partial loss of movement (paresis) on the left side of the body.
^b =^ Left-sided hemiplegia is characterized by paralysis or severe weakness on the left side of the body.

| **Table 3 Section C.** Descriptive statistics for ball bounce speed. | | | | |  |
| --- | --- | --- | --- | --- | --- |
|  | | **Patient A** | **Patient B** | |  |
| Patient Sessions |  | 12 |  | 12 |  |
| Median (s)* |  | 1.199 |  | 1.201 |  |
| Mean (s) |  | 1.199 |  | 1.199 |  |
| Std. Deviation (s) |  | 0.003 |  | 0.003 |  |
| IQR (s) |  | 0.007 |  | 0.007 |  |
| Variance (s^2^) |  | 1.002×10^-5^ |  | 1.069×10^-5^ |  |
| Minimum (s) |  | 1.195 |  | 1.195 |  |
| Maximum (s) |  | 1.202 |  | 1.202 |  |

*(s) = seconds

Ball bounce speed may be considered a proxy for task difficulty, as faster bounce intervals require quicker visuomotor responses and increased motor planning demands, thereby increasing the challenge of the task (see e.g., [100,101]). To ensure consistency across sessions and between participants, bounce speed was kept approximately constant. This design choice allowed for clearer interpretation of user engagement and performance outcomes without the confounding effect of variable task difficulty.

As indicated in the supplementary information, Table S3, ball bounce speed remained stable across all sessions for both Patient A and Patient B, with a mean and median of approximately 1.199 seconds. The standard deviation was minimal (0.003 s), and both the interquartile range (0.007 s) and variance (∼1.0 × 10⁻⁵ s²) indicate very low variability. The identical minimum (1.195 s) and maximum (1.202 s) values across patients confirm tight control over task difficulty throughout the intervention.

**Supplementary Information Section A – HTC Vive Head Mounted Display**

The HTC Vive HMD is a VR device equipped with a pair of high-resolution Organic Light Emitting Diode (OLED) displays, one for each eye, providing a combined resolution of 2160 x 1200 pixels. The HTC Vive utilizes a tracking system known as Lighthouse tracking. This technology uses two base stations that emit infrared lasers, enabling the HMD and accompanying controllers to be tracked. The HTC Vive HMD connects to a computer via a tether, intended for minimal latency. It interfaces with the computer through High-Definition Multimedia Interface (HDMI), Universal Serial Bus (USB), and power connectors. Integrated audio is provided through built-in headphones, reducing the need for external headsets and enhancing the overall VR experience. The HTC Vive supports room-scale VR experiences, allowing users to move within a defined physical space and interact with the virtual world, due to its tracking system and sensors. Systems based on HRTFs require head-tracking when using headphones, which is well suited for the HTC Vive HMD as it uses an HMD paired with headphones [102].

**Supplementary Information Section B – Leap Motion Controller**

The Leap Motion Controller is an optical hand tracking module that can be attached to the HTC Vive HMD using a USB cable. It utilizes an infrared camera to continuously capture high-resolution images at a rapid frame rate, allowing for real-time hand and finger movement tracking within its effective range of about 8 cubic feet[64]. Notably, the Leap Motion Controller establishes no physical contact with the user's hands; it operates entirely through visual input. While it can accurately interpret and render hand gestures and finger positions [64] within the virtual environment, it does not provide haptic feedback, which distinguishes it from other VR input devices that offer tactile responses to interactions. We did not utilize haptic feedback due to the potential complexity it has added when introduced into previous tasks used in post-stroke rehabilitation [65]. This feature positions the Leap Motion Controller as a touchless interface, facilitating intuitive hand-based interactions in VR without the need for handheld controllers.

**Supplementary Information Section C – Description of Patient Test Sessions**

The physiotherapy interactions were standardized to ensure consistency while accommodating individual patient needs. Both physiotherapists underwent uniform training, including an hour-long session on the VR task, supported by developer instructions and feedback from trainee physiotherapists. Sessions followed a consistent structure: patients were introduced to the task with identical instructions and participated in preparatory sessions to familiarize themselves with the VR setup. Physiotherapists maintained uniform task parameters, such as a 1.19-second ball bounce period and consistent use of audio cues during trials. This approach aligns with best practices in VR-based rehabilitation, emphasizing procedural integrity and a patient-centered framework [85].

Specifically, Patient A and Patient B underwent their respective physiotherapy sessions across different dates and were under the care of two distinct, fully certified physiotherapists. Each therapist received one hour of training using the VR task, and were also given instructions provided by the developer, as well as insights shared by physiotherapy trainees who responded to the survey. Prior to both patients commencing their physiotherapy, the physiotherapists introduced the VR task to both the patients, explained the task's objectives, and provided detailed instructions on how to complete it. The task was completed by both patients, first by sitting in a chair and then standing upright with the support of their physiotherapists. Subsequently, both participants were given an opportunity to practice and familiarize themselves with the VR task during a preparatory session. In addition, this session provided both patients an opportunity to familiarize themselves with the apparatus that they would have to interact with; this specifically included the HMD. Notably, Patient A had no prior experience using VR, while Patient B had used VR during previous physiotherapy training. Neither patient experienced any 3D motion sickness. Both physiotherapists would assist each patient with fitting the HMD to their head before adjusting to comfort. The presence of the physiotherapists was essential for manually initiating each VR trial, as they selected the “start” option from the in-game menu (as depicted in supplementary figure S2, illustrating the in-task menu the physiotherapists interacted with prior to commencing each trial). An average ball bouncing period (trajectory of up-down speed of the ball) of 1.19s was selected for both patients by their physiotherapists. In addition, audio cues were also used during these test trials. Both physiotherapists monitored the sessions in real time using a secondary display device with headphones that mirrored the patient’s HMD view and audio. They provided immediate verbal feedback based on each patient’s functional status, rehabilitation goals (see Tables 1, *Physiotherapy goals* and 3, *Assessment of Upper Limb Motor Function*), and observed in-game actions, including compensatory motor strategies such as trunk rotation, delayed reaching, or head turning to initiate gaze shifts toward the neglected hemispace. For example, when a patient exhibited hesitation in initiating a grasp, therapists prompted postural adjustment or encouraged spatial orientation through head or trunk movement. Feedback was adapted across sessions in response to individual motor behavior and task performance. All therapist-patient interactions were confidentially documented in session notes.

# **Supplementary Information Section D – Activities of Daily Living / Barthel Index**

​​**ADL-toiminnat/ Barthelin indeksi:**

| **Toiminta** | **Toimintakyky** | **Pisteet** |
| --- | --- | --- |
| Ruokailu | Täysin autettava | 0 |
|  | Osittain autettava, esim. ruoan leikkaus | 5 |
|  | Itsenäinen | 10 |
| Siirtyminen vuoteesta tuoliin | Vuodepotilas | 0 |
|  | Pystyy istumaan, täysin autettava siirroissa | 5 |
|  | Tarvitsee vähän apua | 10 |
|  | Itsenäinen | 15 |
| Siisteys: hiukset, hampaat ym. | Tarvitsee apua | 0 |
|  | Itsenäinen | 5 |
| WC:ssä käynti | Täysin autettava | 0 |
|  | Tarvitsee vähän apua | 5 |
|  | Itsenäinen | 10 |
| Kylpeminen | Tarvitsee apua | 0 |
|  | Itsenäinen | 5 |
| Liikkuminen | Vuodepotilas | 0 |
|  | Vain pyörätuolilla 50 m | 5 |
|  | Tarvitsee apua, kävelee 50 m | 10 |
|  | Kävelee itsenäisesti 50 m    Apuvälineet: ____________________________ | 15      ________ |
| Kävely portaissa | Ei | 0 |
|  | Tarvitsee apua | 5 |
|  | Itsenäinen | 10 |
| Pukeutuminen ja riisuutuminen | Täysin autettava | 0 |
|  | Tarvitsee apua | 5 |
|  | Itsenäinen | 10 |
| Suoli | Täysin inkontinentti | 0 |
|  | Ajoittain inkontinentti | 5 |
|  | Normaali | 10 |
| Rakko | Täysin inkontinentti tai katetri | 0 |
|  | Ajoittain inkontinentti, ei katetria | 5 |
|  | Kontinentti | 10 |
| 10/09/TU | Kokonaispistemäärä ____ / | max 100 p |
